# MR-guided reconstruction of PET data in spinal cord PET/MRI

**DOI:** 10.3389/fnume.2025.1706155

**Published:** 2025-12-11

**Authors:** Eve Lennie, Charalampos Tsoumpas, Nigel Hoggard, Thomas Jenkins, Matthew Spangler-Bickell, Steven Sourbron

**Affiliations:** 1 Department of Clinical Medicine, University of Sheffield, Sheffield, United Kingdom; 2 Department of Nuclear Medicine and Molecular Imaging, University Medical Center Groningen, University of Groningen, Groningen, Netherlands; 3 Department of Radiology, Sheffield Teaching Hospitals NHS Foundation Trust, Sheffield, United Kingdom; 4 Joondalup Healthcare Campus and Curtin University, Perth, WA, Australia; 5 PET/MR Engineering, GE HealthCare, Waukesha, WI, United States

**Keywords:** PET/MRI, positron emission tomography, magnetic resonance imaging, spinal cord, neurology, neuro-imaging, quantification

## Abstract

**Introduction:**

Accurate PET reconstruction in spinal cord PET/MRI is challenging due to the small size of the structure and interference from background activity. The aim of this study was to establish whether MR-guided PET reconstruction can improve the accuracy of measured uptake in the spinal cord.

**Methods:**

The hybrid kernel expectation maximisation (HKEM) algorithm was evaluated on a digital anthropomorphic phantom (XNAT), and an implementation of a modified asymmetric Bowsher's prior incorporating both PET and MR data was evaluated on clinical test cases. The methods were compared against commonly used algorithms OSEM and Q.Clear.

**Results:**

The results demonstrated that the two algorithms lead to an increase in measured [_18_*F*]FDG PET tracer uptake in the spinal cord. Comparison to ground truth indicates that the improvement is insufficient to remove the bias in this small structure.

**Discussion:**

With care taken to optimise for the desired application, novel PET image reconstruction algorithms using PET and MR data to inform iterative image updates lead to improved quantification and improved image quality compared to OSEM. Further work is needed to investigate the optimal parameters and identify strategies to reduce residual bias.

## 1 Introduction

Anatomically guided PET reconstruction is a longstanding field of research in medical image reconstruction (1), with the algorithms made more feasible by the widespread use of combined PET/MR scanners. MRI can provide high resolution anatomical images with high contrast between different soft tissue structures, which can be utilised by MR-guided PET image reconstruction algorithms to improve localisation of PET activity and resolution recovery in PET images.

Several approaches have been developed for anatomically guided PET reconstruction which include anatomical information into an iterative reconstruction technique. The maximum *a posteriori* expectation maximisation (MAP-EM) algorithm (2) can be modified to include anatomical information from MR as a prior (3). Bowsher et al proposed a method to incorporate an anatomical prior into bayesian reconstruction algorithms (4) by computing edge information from an anatomical image to avoid over-smoothing across edges by the penalisation factors. This approach is popular, and has since been applied to MAP-EM reconstruction (5, 6). Joint entropy (JE) or mutual information (MI) approaches (7) devise a similarity weighting between PET and MR information to further guide the penalty function in bayesian reconstruction methods, making the algorithm more robust to mismatches between PET and MRI. Finally, kernel expectation maximisation (KEM) (8) and the hybrid kernel expectation maximisation (HKEM) (9) incorporate anatomical information into the more familiar maximum likelihood (ML) iterative algorithm by constructing a kernel matrix prioritising similarity between the image update and the kernel matrix.

Simultaneous acquisition of PET and MR improves spatial co-registration of images and reduces errors in anatomically guided image reconstruction (1, 10), while the inclusion of both PET and MR information into the image reconstruction process further reduces the impact of image misalignment between PET and MR (11, 12). These methods have been shown to outperform partial volume correction applied post-reconstruction (5). Many of these algorithms are demonstrated for use in brain PET/MRI (3, 5, 6, 8, 13), however, conditions that affect the wider central nervous system (CNS) such as Multiple Sclerosis (MS) and Amytrophic Lateral Sclerosis (ALS) warrant interest in imaging the spinal cord (14, 15), particularly as more CNS specific tracers continue to be developed (16). The HKEM algorithm (9) appears promising for use in the spinal cord as it has previously been shown to improve the image quality for PET images of the carotid arteries (9) and aortic aneurysms (17), which are both small structures in areas of relatively high background activity.

The aim of this study was to establish whether using MR-guided PET reconstruction algorithms can improve the accuracy of measured uptake in the spinal cord, when compared to commonly used algorithms OSEM and Q.Clear without MR guidance. Our secondary aim is to determine whether MR-guided PET reconstruction leads to an improvement in PET image quality compared to OSEM and Q.Clear reconstructed images. We present the first results on MR-guided reconstruction in spinal cord imaging in PET/MRI (18), using open-source methods on simulated data and a commercial method on patient data.

## 2 Methods and materials

### 2.1 Theory

The HKEM algorithm allows for anatomical information to be introduced to the model based algorithms by using a kernel matrix to represent the features and allow the problem to be treated as linear. In PET/MR image reconstruction, the kernel matrix has a PET and an MR component. The kernel is defined as Equations 1–3:

$$k_{lj}^{\left(n\right)}=k_{m}(v_{l},v_{j})\cdot k_{p}(z_{l}^{\left(n\right)},z_{j}^{\left(n\right)})$$ (1)

with the MR component being:

$$k_{m}(v_{l},v_{j})=\exp\left(-\frac{\|v_{l}-v_{j}{\|}^{2}}{2\sigma_{m}^{2}}\right)\exp\left(-\frac{\|x_{l}-x_{j}{\|}^{2}}{2\sigma_{\mathrm{dm}}^{2}}\right)$$ (2)

and the PET component:

$$k_{p}(z_{l}^{\left(n\right)},z_{j}^{\left(n\right)})=\exp\left(-\frac{\|z_{l}^{\left(n\right)}-z_{j}^{\left(n\right)}{\|}^{2}}{2\sigma_{p}^{2}}\right)\exp\left(-\frac{\|x_{l}-x_{j}{\|}^{2}}{2\sigma_{\mathrm{dp}}^{2}}\right)$$ (3)

where $\sigma_{p}$, $\sigma_{m}$, $\sigma_{\mathrm{dp}}$ and $\sigma_{\mathrm{dm}}$ are scaling factors for the strength of each component of the prior, and the second Gaussian in each component acts on positional vectors $x_{l}$ and $x_{j}$ so that voxels must not only be similar in features, but also close range enough to be considered correlated voxels. This has been shown to preserve PET unique features better than the initial KEM implementation even before the addition of the PET kernel (19). The matrix form can then be used to create a kernel based projection model for use in EM approaches to PET image reconstruction as (Equation 4)

Y=AKα+S+R (4)

### 2.2 Simulation

The XCAT mathematical phantom (version 2) (20) was used to generate [^18^*F*]FDG tracer distributions of organs in the neck and thorax for a single 25 cm field of view based on reported uptake in healthy subjects (21–25). We used the XCAT standard male and standard female phantoms. Phantoms were simulated to a voxel size of $2.1\times 2.1\times 2.8\;{\mathrm{mm}}^{3}$. 511 keV photon attenuation maps were also generated for the region by the XCAT software. Attenuation maps were scaled to units ${\mathrm{cm}}^{-1}$. Modified attenuation maps were also generated to simulate those derived from Dixon MRI sequences, which was achieved by replacing all bone linear attenuation coefficients $\geq 1.2\;{\mathrm{cm}}^{-1}$ with a muscle linear attenuation coefficient of 0.99 ${\mathrm{cm}}^{-1}$ (26). Activity in the spinal cord was set to a constant value of 8.75 kBq/ml in the male XCAT phantom, and 8.5 kBq/ml in the female phantom.

An anatomical MR image of the XCAT phantom was simulated by assigning pixel intensity values for major tissue types in T2-weighted MR images, as measured from a sagittal T2-weighted FSE image acquired on-site, to the XCAT phantom in place of organ activity values for the spinal cord, bone marrow, cortical bone, and lung, then assigning a single fat or muscle image pixel intensity to all other organs and tissues within the field of view. A prior with just the spinal cord segmented from the synthetic MR was also created for each phantom.

To perform simulations at a scanner detector resolution representative of a clinical PET/MRI scanner, the average distance of the spinal cord to the image centre was measured on patient acquisitions so that NEMA performance results for the scanner could be used to determine an appropriate resolution for our simulation representative of spinal cord acquisitions. From an average distance of 2.4 cm, a transaxial resolution of 4.4 mm and an axial resolution of 6 mm, which was simulated by applying a 3D Gaussian filter to the generated XCAT activity distributions and attenuation maps using ImageJ (27).

Each XCAT distribution was forward projected using SIRF (version 3.4.0) (28) to generate a sinogram of the distribution. Attenuation correction factors (ACFs) were obtained from the attenuation maps with bone attenuation coefficients present, and scatter was calculated using the Single Scatter Simulation (SSS) algorithm in STIR (version 5.0.2) (29). The XCAT activity, ACF and scatter sinograms were combined for sinograms simulating acquired PET data (30). Noise was added to sinogram data by randomly drawing samples from a Poisson distribution. The number of counts in the sinogram was scaled to equal an average value measured in the same field of view of both patient data. The sinogram was then scaled back to the original number of counts prior to image reconstruction. Time of flight information was not included in simulated data.

ACFs and scatter were also calculated for the attenuation maps without bone to be used during image reconstruction. Simulated sinograms were reconstructed using an Ordered Subset Expectation Maximisation (OSEM) algorithm (28 subsets, 10 iterations, voxel size $2\times 2\times 2.8\;{\mathrm{mm}}^{3}$) with attenuation and scatter correction. Image reconstruction for each phantom was performed twice: once with attenuation and scatter correction calculated from the attenuation map with bone and once with corrections calculated from the attenuation map without bone. Point spread function (PSF) modelling was not included. A 5 mm Gaussian filter was applied post-reconstruction as this is often used in the clinical setting.

HKEM image reconstruction (28 subsets, 10 iterations, voxel size $2\times 2\times 2.8\;{\mathrm{mm}}^{3}$) was performed with the simulated T2-weighted MR image provided as a prior for the reconstruction kernel and uses the attenuation map without bone features for attenuation and scatter correction. To determine whether the prior should have the organ of interest segmented out first, reconstructions were also performed using just the spinal cord segmented from the synthetic T2 MRIs. This was assessed as in some previous work, the organ of interest was segmented MR images prior to supplying to the MR kernel (9). Parameters for the kernel used were varied to determine the optimal parameters for most uptake measured. $\sigma_{\mathrm{dm}}=3$, $\sigma_{\mathrm{dp}}=3$ where kept consistent, but $\sigma_{m}=0.1,1$, $\sigma_{\;p}=0.1,0.5,1$, where $\sigma_{m}$ and $\sigma_{\mathrm{dm}}$ are scaling factors for the MR part of the kernel and $\sigma_{\;p}$ and $\sigma_{\mathrm{dp}}$ are scaling factors for the PET part. The HKEM algorithm operates over an N×N voxel neighbourhood of the input images, and neighbourhood size N=3,5 were tested for their impact on reconstructed images. HKEM was not filtered separately as the algorithm is designed to reduce noise in the reconstruction.

Spherical Regions of interest (ROIs) of 5 mm diameter were drawn in the spinal cord at each vertebral level corresponding to vertebra C1 to T5. Mean activity and standard deviation were measured for each ROI.

Contrast to noise ratio (CNR), Coefficient of Variation (CoV) and bias were used as image quality metrics. CNR is calculated as Equation 5

$$\mathrm{CNR}=\frac{s-b}{\sqrt{{\mathrm{SD}}_{s}^{2}+{\mathrm{SD}}_{b}^{2}}}$$ (5)

where s is the mean value in the spinal cord ROI, b is the mean value in the reference region. ${\mathrm{SD}}_{s}$ and ${\mathrm{SD}}_{b}$ are the standard deviation in the spinal cord ROI and the reference region respectively. CoV is Equation 6

$$\mathrm{CoV}=\frac{\sigma }{\mu }\times 100$$ (6)

where σ is the ROI standard deviation and μ in the ROI mean. CNR and CoV were averaged across all spinal cord ROI. A 10 mm ROI in the aortic arch was used for the reference region. Wilcoxon signed rank test was used to determine the statistical significance of results, as this analysis is suitable for non-parametric paired data. Results are considered statistically significant where P<0.05.

Bias was computed as the relative difference with the ground truth activity values Equation 7:

$$\mathrm{bias}=100\;*\;\frac{s-s_{\mathrm{truth}}}{s_{\mathrm{truth}}}$$ (7)

### 2.3 Clinical acquisition

Imaging was performed on the SIGNA PET/MR scanner (GE HealthCare, WI, USA) in accordance with the Declaration of Helsinki, with ethics committee approval and all participants gave written informed consent. Two participants, a healthy volunteer and an ALS patient, were administered 250 MBq [^18^*F*]FDG bolus injection 60 min before acquisition. PET data was acquired at two bed positions for 10 min each in head-first supine orientation. MRI was performed simultaneously to PET using the body coil for the dedicated attenuation correction Dixon and Zero Echo Time (ZTE) sequences, as well as the following anatomical sequences using a head and neck coil: axial T1-weighted Fast Spin Echo (FSE) and Axial T2-weighted FLAIR (Fluid Attenuated Inversion Recovery) for the brain, and sagittal T2-weighted FSE, Sagittal T1-weighted FLAIR of the spinal cord.

PET image reconstruction was performed offline using the vendor-provided software Duetto version 02.19 using an MR guided list-mode reconstruction algorithm with TOF Q.Clear. This algorithm is an implementation of a modified asymmetric Bowsher’s prior (4) incorporating both PET and MR data through the calculation of a similarity coefficient between the PET and MR images, and incorporated into the existing Bayesian penalised likelihood reconstruction algorithm, Q.Clear (31). The penalisation factor for MR guided reconstruction, μ was set to 100. An initial PET seed is reconstructed with OSEM (30 subset, 1 iteration) for use as an additional prior and the Sagittal T1- and T2-weighted spine MR images are used to generate a similarity coefficient for assigning voxel neighbourhoods. Subsequent image updates apply a penalty for noise suppression over pixel neighbourhoods defined using the similarity weighting between PET and MR anatomical images. Areas of the PET field of view for which no anatomy is provided are not penalised by the MR guided algorithm parameters. Reconstructions were also performed using the sinogram-based TOF Q.Clear algorithm for comparison, with b = 0, 100, 200, and 400, all of which are initialised using a 2 iteration OSEM reconstruction. Both of these algorithms include PSF correction.

Activity is normalised to body weight and displayed as Standardised uptake values (${\mathrm{SUV}}_{\mathrm{bw}}$), which is used in all results presented for this part of the study. Spherical ROIs of 5 mm diameter were drawn in the spinal cord at each vertebral level on the T2 weighted MRI from C1 to T6 and used to mean SUV (${\mathrm{SUV}}_{\mathrm{mean}}$) and standard deviation for each ROI in the PET images.

${\mathrm{SUV}}_{\mathrm{bw}}$ was averaged over the datasets. Wilcoxon signed rank test was used to determine the statistical significance of results. CNR and CoV were also calculated using a reference region in the aortic arch.

## 3 Results

### 3.1 XCAT simulations

**Figure 1:**
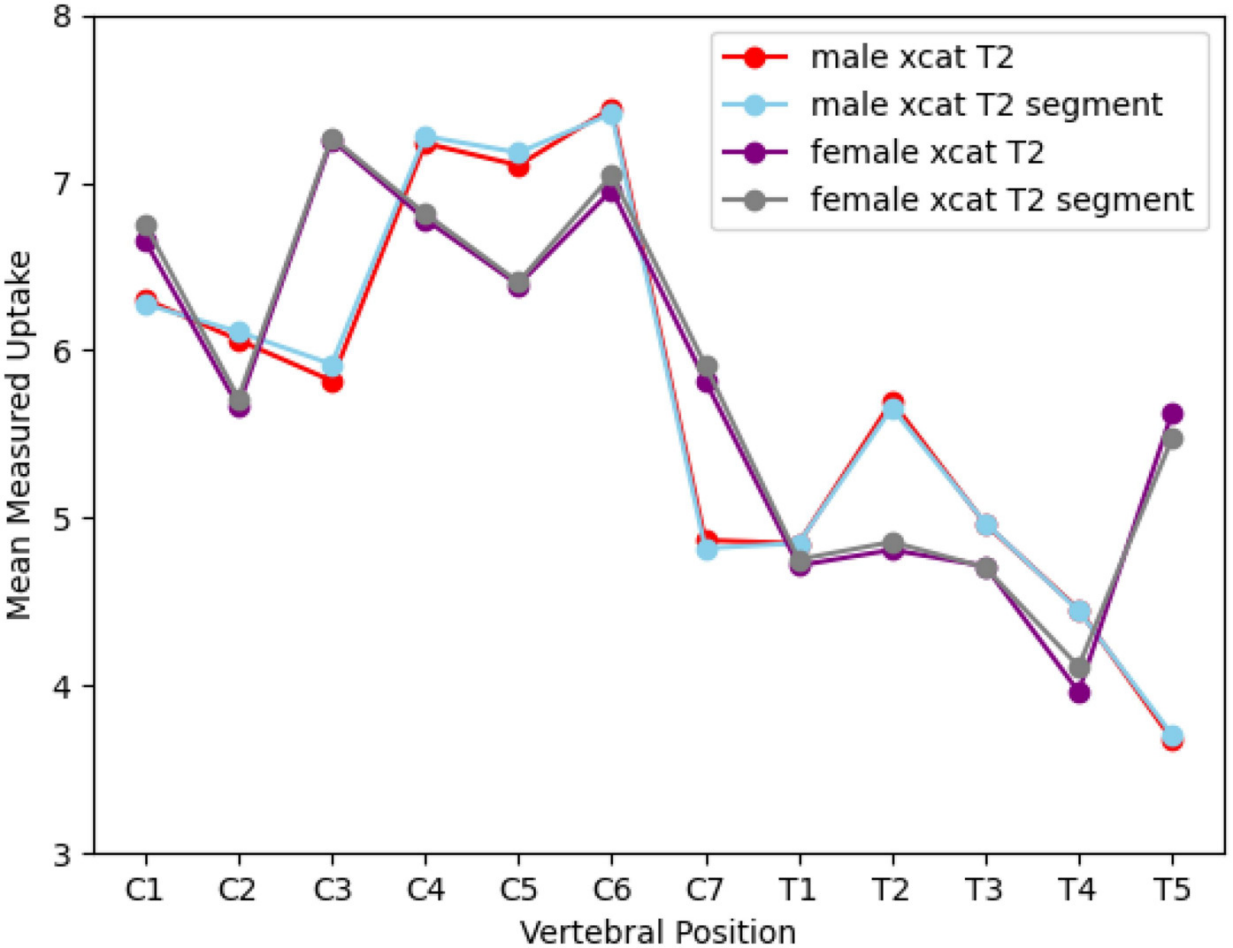
A graph showing the effect of different T2 MR priors, where “segment” corresponds to priors for which the spinal cord is segemented out of the MR image, on measured PET uptake in the spinal cord for the male and female XCAT phantoms.

**Figure 2:**
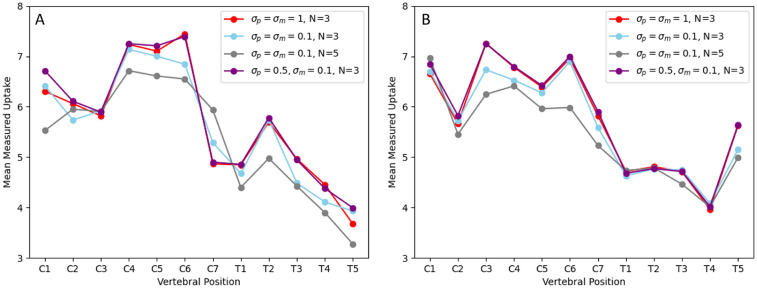
Graphs showing the effect of different HKEM parameters on measured PET uptake in the spinal cord for the male **(A)** and female **(B)** XCAT phantoms.

No difference was found between using the synthetic T2 MR as a prior compared to segmenting out the spinal cord first, as show in Figure 1. Graphs showing the effect of different HKEM parameters on uptake measurements for the XCAT phantoms are displayed in Figure 2. Changing $\sigma_{m}=\sigma_{\;p}=0.1$ reduced uptake measured at some vertebral positions compared to $\sigma_{m}=\sigma_{\;p}=1$, but made no significant difference to results (Male XCAT phantom p=0.3, female XCAT phantom p=0.06). Similarly, setting $\sigma_{\;p}=0.5$ with $\sigma_{m}=0.1$ made no apparent difference to uptake measurement compared to $\sigma_{m}=\sigma_{\;p}=1$ (Male XCAT phantom p=0.09, female XCAT phantom p=0.06). Therefore, $\sigma_{m}=\sigma_{\;p}=1$ was chosen as the optimal HKEM reconstruction values for comparison with OSEM in line with previously reported results (17). Increasing the number of voxels in the voxel neighbourhood *N* from 3 to 5 reduced measured uptake along the length of the spinal cord (Male XCAT phantom p=0.04, female XCAT phantom p=0.02). This is to be expected, as this permits smoothing over a large area of voxels, which improves image quality metrics whilst smoothing the signal intensity over the larger neighbourhood. As a result, N=3 was chosen as the optimal value for our HKEM reconstructions.

**Figure 3:**
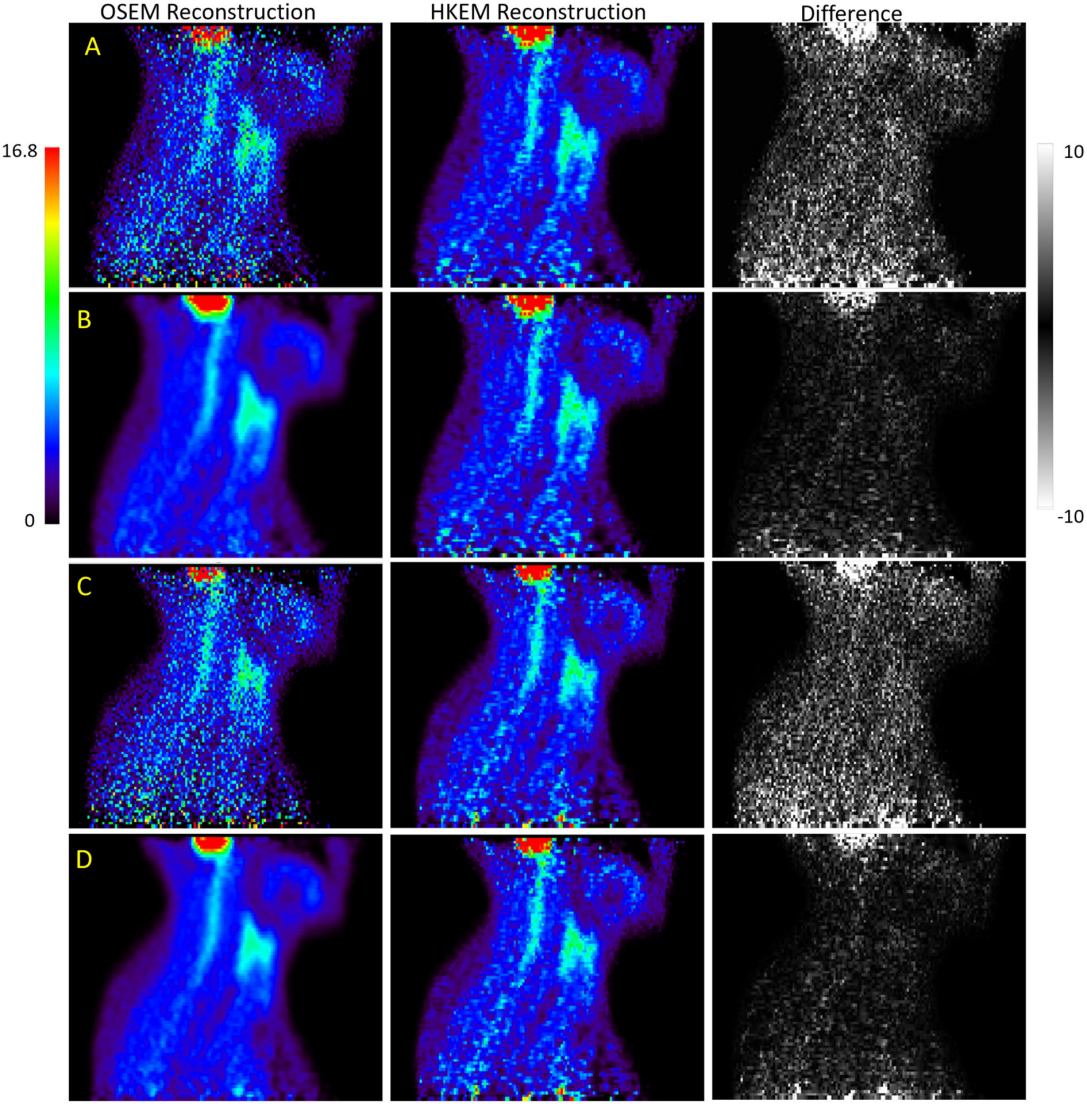
Images of the XCAT male [rows **(A,B)**] and female [rows **(C,D)**] phantoms reconstructed with OSEM (first column) and HKEM (second column). The first column in rows **(B)** and **(D)** show post-filtered reconstructed OSEM images. The HKEM images in rows **(B)** and **(D)** were not filtered. The last image in each row shows the difference between OSEM and HKEM reconstruction.

Images of both XCAT phantoms with OSEM and HKEM algorithms, and difference images are presented in Figure 3. HKEM reconstructions appear markedly different to OSEM reconstructions, particularly in noise present across the entire image, which is shown in rows A and C of Figure 3. However, when compared to post-filtered OSEM reconstructions, the difference between images is reduced and is predominantly in the areas of the brain, with slight difference visible in the spinal cord (rows B and D of Figure 3).

**Figure 4:**
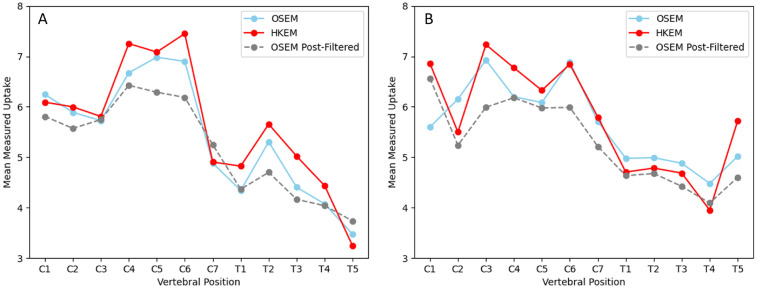
Graphs showing measured uptake along the spinal cord for the male xcat phantom **(A)** and female xcat phantom **(B)** in OSEM, Post-filtered (5 mm Gaussian) OSEM and HKEM images. All images are attenuation and scatter corrected using an attenuation map without bone, simulating an MR derived attenuation map.

Analysis of measured activity uptake in the spinal cord showed an average increase in measured uptake of 3.9% in the HKEM male XCAT phantom image compared to OSEM, and a maximum of 12% increase at T3, visible on graph A in Figure 4. Differences were statistically significant with a *p*-value of p=0.03. Image quality metrics are displayed in Table 1, and are improved in the HKEM image compared to OSEM. All values were severely underestimated compared to the ground truth but the difference in bias between the methods is relatively small (Bias from −42% to −35%). Post-filtering reduces the variability substantially at the cost of a small additional bias.

**Table 1:** Image quality metrics in reconstructed PET images of the XCAT phantoms for OSEM, Post-filtered OSEM and HKEM algorithms, for ROIs in the spinal cord of male (M) and female (F) XCAT phantoms.

Reconstruction algorithm	CNR		CoV		Bias	
	M	F	M	F	M	F
OSEM	0.3	0.6	46%	36%	−38%	−35%
OSEM + Post filter	2.2	3.9	5%	5%	−42%	−39%
HKEM	0.9	1.5	18%	16%	−35%	−32%

In the female XCAT phantom, the average increase in measured uptake is overall much smaller, with an average increase of 0.7% in measured uptake in the HKEM image, despite the larger maximum increase of 18.4% at C1. This is shown in graph B of Figure 4, which also demonstrates that in the female phantom, measured uptake in the HKEM is generally increased in the cervical spine compared to OSEM, but decreased in the thoracic spine (p=0.62). Image quality metrics are also improved in the HKEM image compared to OSEM for the female XCAT phantom.

Results for post-filtered OSEM reconstructed images are displayed as a dashed line in Figure 4. When post-filtering is applied to OSEM images, measured uptake is reduced compared to both OSEM without post-filtering and HKEM reconstructed images in both phantoms. In the male phantom, measured uptake in the HKEM reconstructed image is an average of 4.6% higher (p=0.02, maximum increased uptake 29.6% at C6) and in the female phantom by an average of 7.4% (p=0.002, maximum increased uptake 19.6% at T5). CNR for the post-filtered OSEM images is lower than the HKEM reconstructed image for the male XCAT phantom (CNR=0.7), but not the female XCAT phantom (CNR=3.9). However, CoV is lower in post-filtered OSEM images the HKEM reconstructed images in both cases (male phantom: CoV=4.4%, female phantom: CoV=4.8%).

**Figure 5:**
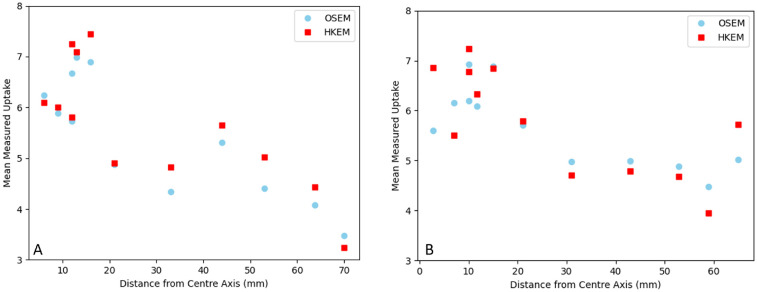
Graphs showing measured uptake in the spinal cord for the male xcat phantom **(A)** and female xcat phantom **(B)** against ROI displacement from the centre of the field of view transaxially.

The spinal cord isn’t at a fixed distance from the isocentre for its full length. As resolution varies across the PET field of view, decreasing with transaxial distance from the isocentre (32), we have shown how uptake changes with ROI displacement from the centre of the field of view in the transaxial plane in Figure 5. Both phantoms show a decrease in measured uptake with increasing distance from the image centre, and in graph A of Figure 5, it appears that HKEM recovers activity well in the distal ROIs, however this is not demonstrated for both cases.

### 3.2 Clinical acquisitions

**Figure 6:**
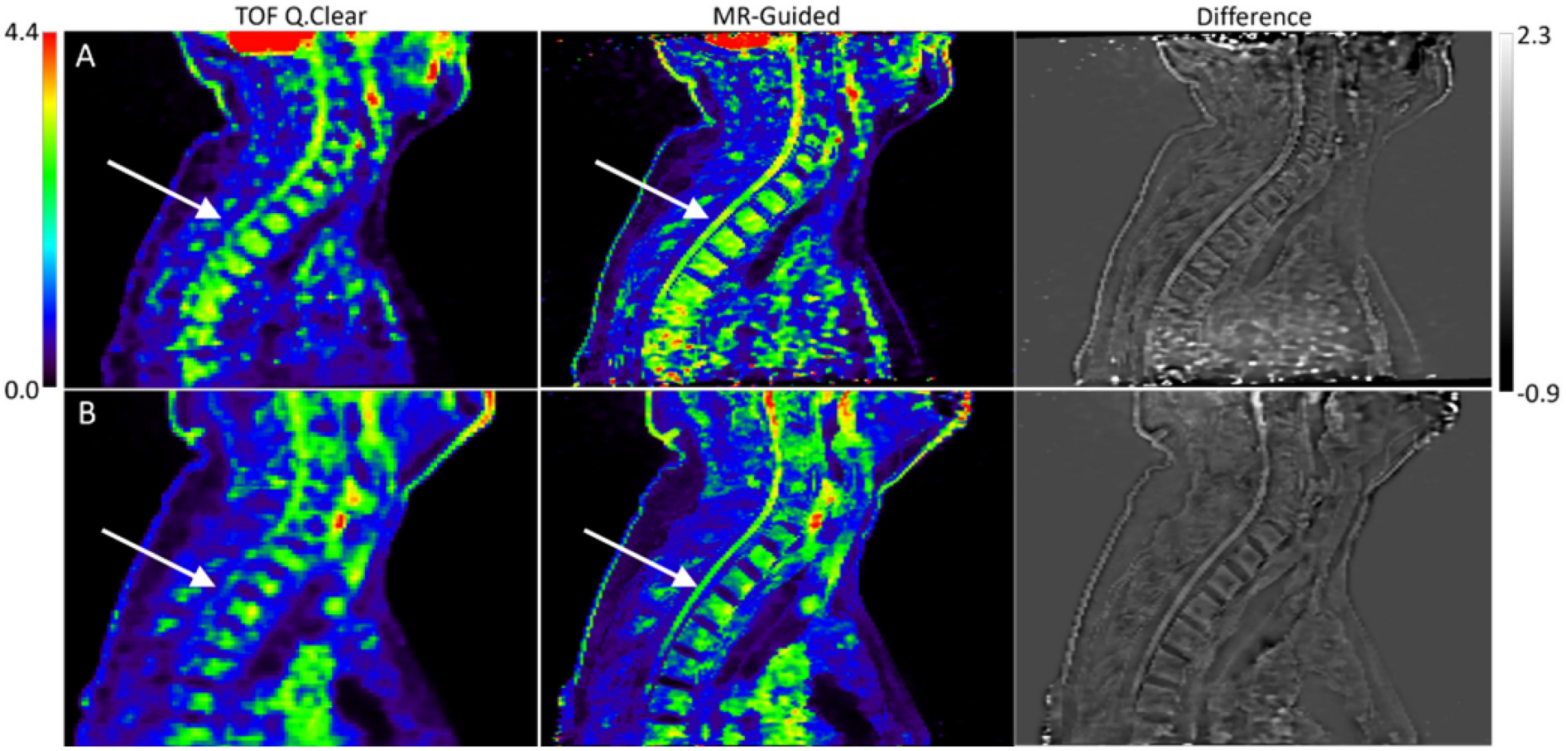
[^18^*F*]FDG PET images of a volunteer [row **(A)**] and Amytrophic Lateral Sclerosis (ALS) patient [row **(B)**] reconstructed with TOF Q.Clear, MR-guided TOF Q.Clear and showing a difference image betweens the reconstruction algorithms. The white arrow indicates the spinal cord in the thoracic spine, more clearly visualised in the MR-guided Q.Clear reconstructed images compared to TOF Q.Clear images.

Images of both clinical subjects reconstructed with TOF Q.Clear and MR-guided TOF Q.Clear are displayed in Figure 6. The MR-guided reconstructed images maintain the noise suppression provided by Q.Clear, but visibly enhance anatomical edges of the spine and spinal cord. As a result, resolving between bone marrow uptake and spinal cord uptake in the thoracic spine is visually clearer in the MR-Guided Q.Clear images shown in the second column of Figure 6.

**Figure 7:**
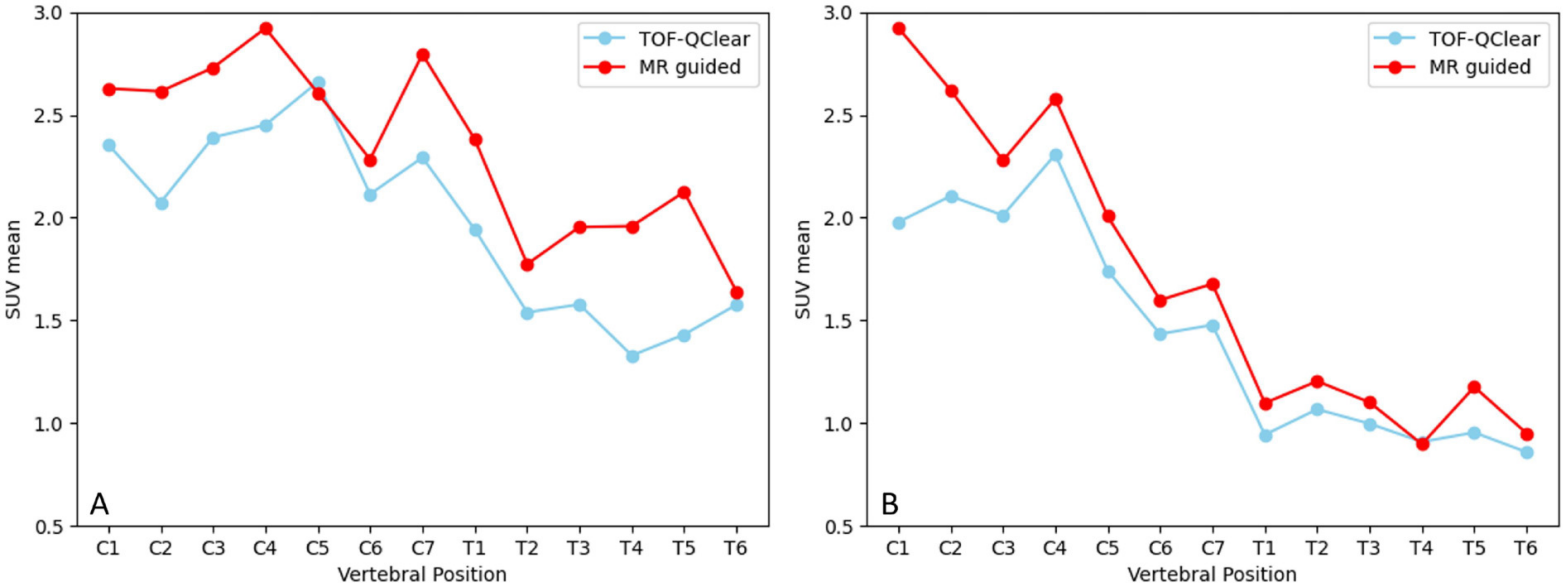
Graphs showing ${\mathrm{SUV}}_{\mathrm{mean}}$ along the spinal cord in a volunteer **(A)** and ALS patient **(B)**, reconstructed with TOF Q.Clear and MR guided TOF Q.Clear algorithms.

**Figure 8:**
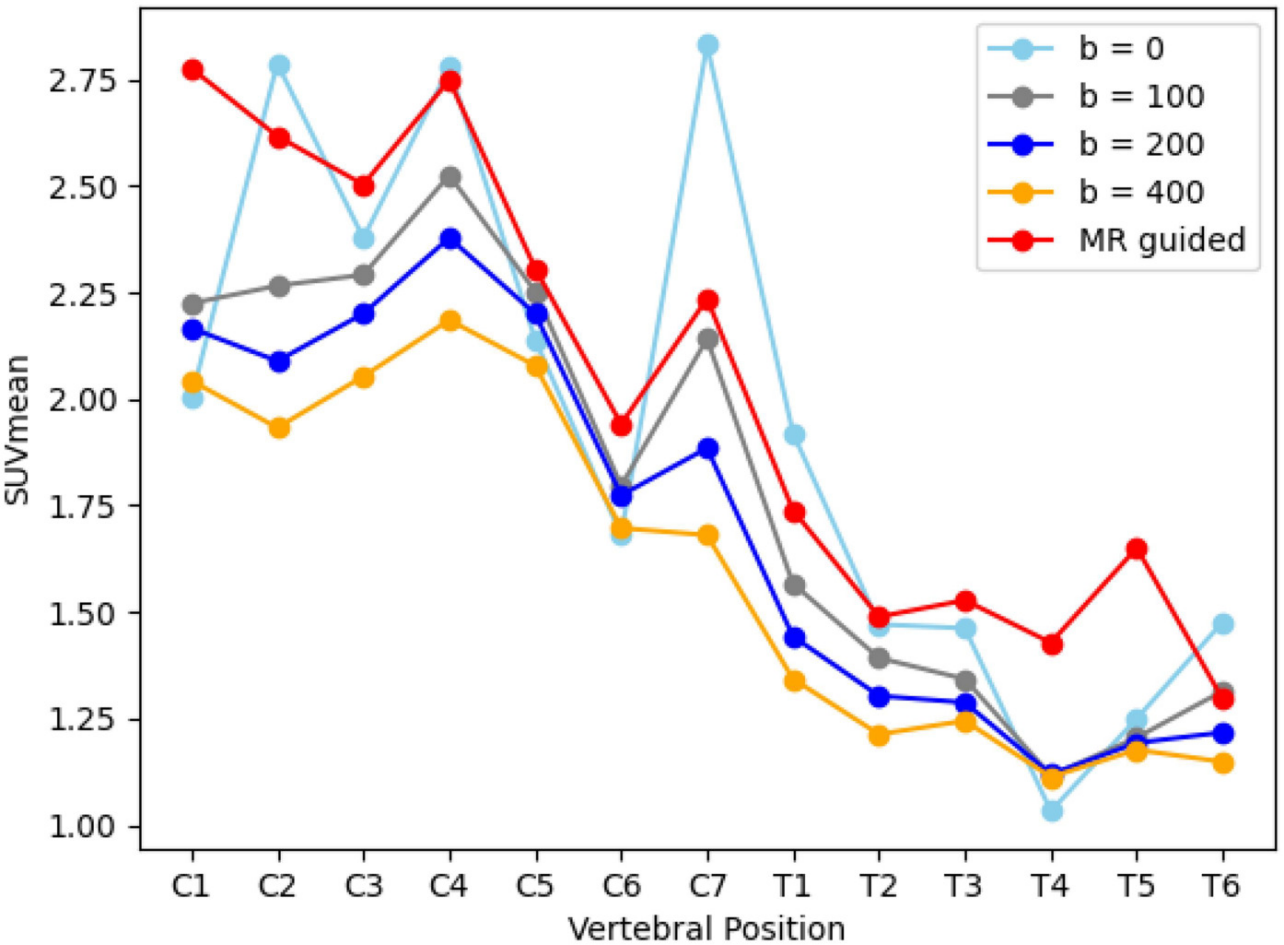
A graph showing the average ${\mathrm{SUV}}_{\mathrm{mean}}$ along the spinal cord when reconstructed with TOF Q.Clear for different b values and MR guided TOF Q.Clear (b=0).

Given the difference in HKEM performance during the simulated study between the male and female phantom presented in Section 3.1, results here are also segregated with graphs showing ${\mathrm{SUV}}_{\mathrm{mean}}$ against vertebral position presented for both subjects in Figure 7. However, in both cases the MR-guided reconstruction shows an increased uptake in the spinal cord when compared to Q.Clear (average increase in measured uptake: 27.1%, p≤0.001 and 50.7%, p=0.03) and TOF Q.Clear (average increase in measured uptake: 24.7%, p≤0.001 and 50.6%, p≤0.001) reconstructed images. This is demonstrated by looking at plots of ${\mathrm{SUV}}_{\mathrm{mean}}$ averaged over both patients in Figure 8, where the higher quantification in MR guided reconstruction is seen compared to TOF Q.Clear for comparable values of beta (p=0.49).

**Figure 9:**
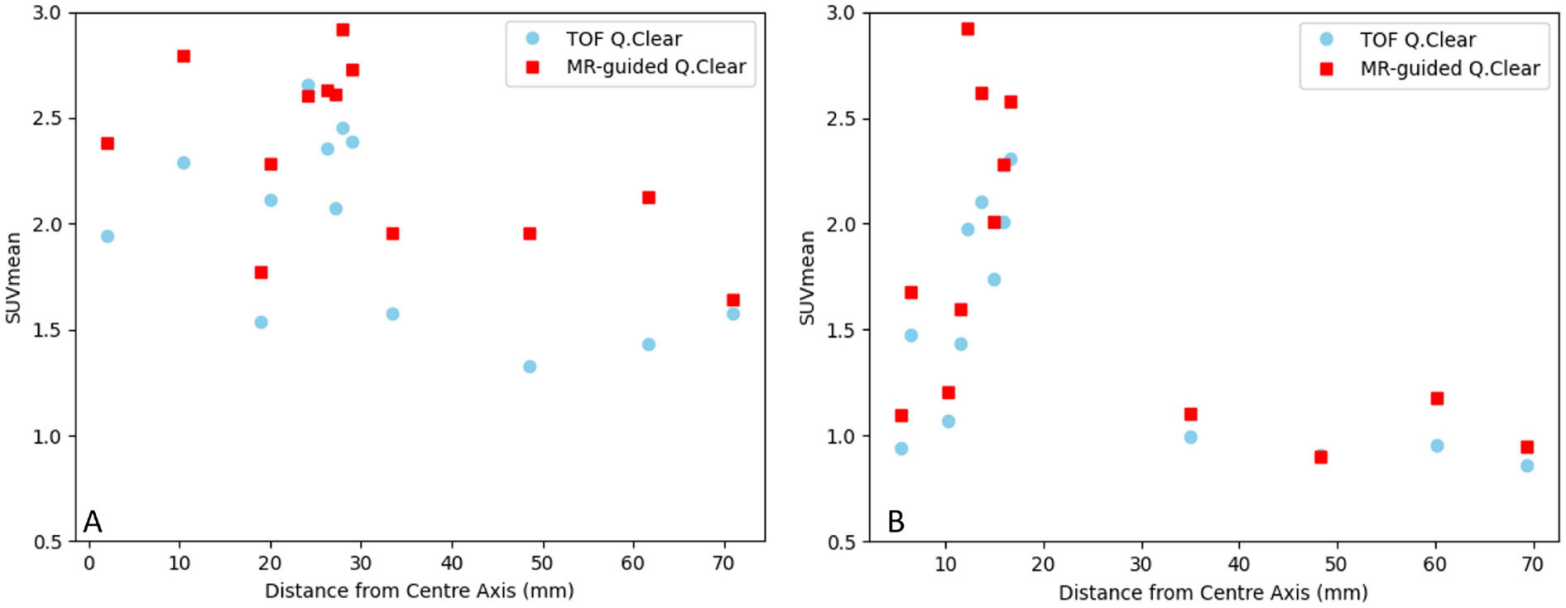
Graphs showing ${\mathrm{SUV}}_{\mathrm{mean}}$ along the spinal cord in a volunteer **(A)** and ALS patient **(B)** against ROI displacement from the centre of the field of view transaxially.

As demonstrated in the XCAT phantom, both the patient and volunteer show a decrease in ${\mathrm{SUV}}_{\mathrm{mean}}$ in ROIs measured further from the centre of the field of view, show in Figure 9. In both cases, MR guided reconstruction is able to recover more activity in distal ROIs than TOF Q.Clear, and this is particularly prominent in graph A of Figure 9.

Only one subject had the reference region within the MR field of view, so image quality metrics are only reported here for those datasets, displayed in Table 2. At an average CNR of 1.94, the MR-guided reconstruction outperforms a comparable TOF Q.Clear with b=0 (CNR = 1.00), but higher b gives a higher CNR. CoV is also higher (CoV = 19%) than TOF Q.Clear with b=0 (CoV = 43%) and comparable to b=100 (CoV = 19%), indicating that MR guidance is reducing image noise.

**Table 2:** Image quality metrics for a clinical acquisition reconstructed with TOF Q.Clear (b=0,100,200) and MR-guided Q.Clear, for ROIs in the spinal cord.

Reconstruction algorithm	Average CNR	Average CoV
TOF Q.Clear (b=0)	1.00	43%
TOF Q.Clear (b=100)	2.32	19%
TOF Q.Clear (b=200)	3.42	12%
MR guided TOF Q.Clear	1.94	19%

## 4 Discussion

### 4.1 XCAT simulations

In optimising the HKEM algorithm for measuring tracer uptake in the spinal cord, we found a smaller voxel neighbourhood for the kernel of N=3 was required compared to previous studies where image quality metrics were prioritised (17). However, other changes to parameters made only small differences to both measured uptake and image quality metrics. As the HKEM algorithm is still establishing use cases, there is a lot to be explored here in balancing MR and PET kernel contributions to each image update given the many possibilities permitted for unequal weighting of the factors.

Improvement to measured uptake compared to OSEM was observed in the male phantom. HKEM also shows a good recovery of activity both near the centre of the field of view, and in more distal ROIs in the transaxial plane, despite a known decrease in detector resolution with distance from the isocentre. In Figure 4 it appears that HKEM underestimates spinal cord activity most in the thoracic spine of the female phantom. This region of the spinal cord has a smaller diameter compared to the cervical spine. No previous patient studies in adults have shown sex differences in spinal cord uptake for [^18^*F*]FDG PET (33), but aspects of the different phantom models and how they are set up could be a factor. For example, uptake of vertebral bone marrow has previously been reported to affect measured spinal cord activity (22) due to its close proximity to the spinal cord. Both XCAT phantoms were assigned organ activity values previously reported in literature (21–25), which leads to the female XCAT phantom having a higher activity assigned to the vertebra and bone marrow than the male phantom, whilst spinal cord uptake is slightly lower. Additionally, the skeletal volume of the spinal column is smaller in the female XCAT phantom (20), so bone marrow is also be closer to the spinal cord.

In our optimised results, post-filtering OSEM reconstructed images gave the highest image quality metrics, though at a small cost in additional bias. On the other hand, our HKEM test with an N=5 voxel neighbourhood size indicate comparable performance to the filter chosen. This highlights the necessity in choosing HKEM reconstruction parameters according to the desired application and is in line with previous studies (9, 17). All methods show substantial bias in the recovered activity, indicating the need for further bias correction in applications where absolute accuracy is important, such as comparisons with reference values, or of results between different devices.

### 4.2 Clinical acquisitions

MR guided PET image reconstruction as implemented in Duetto, and with the parameters used in this study, gives an increased ${\mathrm{SUV}}_{\mathrm{mean}}$ in the spinal cord compared to the currently implemented TOF Q.Clear algorithm. Given the results demonstrated in the simulation section of this study, it can be inferred that the increased uptake measured in the MR-guided reconstructions represent an increase in accuracy towards measuring true uptake. In the graph A of Figure 9 MR-guided reconstruction showed a greater increase in ${\mathrm{SUV}}_{\mathrm{mean}}$ for distal ROIs, which could be attributed to resolution recovery by inclusion of the MR prior, as counts further from the PET isocentre are imaged with lower intrinsic resolution (32).

The edge preservation mechanism creates images that appear sharper, however there is a risk of creating an enforced edge where PET activity crosses the boundaries of MR features (11). Generally, this would not be expected in spinal cord imaging due to low uptake of [^18^*F*]FDG in cerebral spinal fluid (CSF) (22), indicating that MR guided reconstruction presents a benefit to spinal cord PET/MR. Here we used parameters largely tested on brain images previously, so additional work is still needed to optimise reconstruction parameters for spinal cord imaging, particularly the weighting of MR and PET priors.

When compared to TOF Q.Clear, CNR and CoV are improved when MR guidance is used for comparable beta value. This means that noise is reduced in the resulting PET images despite increasing sharpness at tissue boundaries, which is beneficial for imaging small structures that can become overly smoothed when reducing noise in PET imaging (34). There is potential to investigate a combination of the choice of beta and mu parameters with different PET and MR prior weightings.

**Figure 10:**
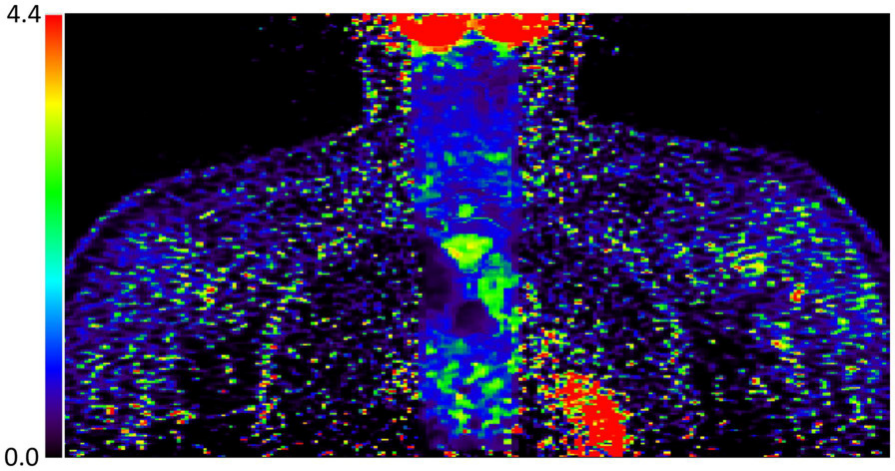
An example of MR guided PET image reconstruction viewed in the coronal plane, showing regions where the MR prior does not cover the full PET field of view. Outside of MR coverage, the PET image is noisy and unclear since these are updated with regular OSEM only.

MR guided reconstruction was applied retrospectively to data that had already been acquired in this study, however, an investigation into the impact that the chosen MR acquisitions have on reconstructions would also be beneficial. Due to having only acquired spinal cord images typical in clinical imaging for Amyotrophic Lateral Sclerosis (ALS) (35), our study used sagittal MRI with low resolution in the axial plane and a field of view restricted to the spine itself. The parameters chosen in this study compromise the PET image outside the MR field of view, which is more apparent when viewing other image planes such as the coronal displayed in Figure 10. Therefore either a large field of view MR sequences would be needed to cover all anatomy, or the weighting of the MR prior may be too high if we are unable to resolve PET features without it. For some applications it may be reasonable to reconstruct both a TOF Q.Clear for the full field of view to assess wider anatomy, whilst using MR guided reconstruction to focus on an organ of interest.

### 4.3 Limitations

Our aim in this study was to evaluate MR guided reconstruction methods in the spinal cord using both patient data and anthropomorphic phantoms. Ideally this would involve applying the same algorithms on both, but due to software compatibility issues we were unable to run open source methods on patient data, or commercial methods on phantoms. We therefore opted to select commercial and open source methods that operate on similar physical principles and use the commercial method on patients and the open source method on digital phantoms. The XCAT data simulated in this study was not uploaded to Duetto for assessment with the MR guided algorithm developed by GE HealthCare. Similarly patient data from the SIGNA PET/MR scanner wasn’t assessed by reconstruction with the HKEM algorithm, as not all required corrections for data import were implemented in the version of SIRF used. This is a limitation that needs to be overcome in future studies so that identical methods can be applied to all data.

Though HKEM and Q.Clear methods operate on similar principles, there are fundamental differences. The HKEM algorithm is an open-source implementation that has previously been validated for other applications (11, 17), and is more suited than commercial methods for basic research, exploration of variables and assessment of bias. MR-guided Q.Clear was assessed as this algorithm is commercially available, is more easily applied to patient data and takes a similar approach to the incorporation of anatomical priors, but it is based on a BSREM image reconstruction algorithm rather than OSEM.

STIR does not have robust PSF modelling for PET data reconstruction, limiting the resolution recovery of reconstructions performed within the framework. It would help to improve partial volume effect by utilising both HKEM and PSF modelling. The GE HealthCare PET Toolbox includes PSF modelling in MR guided PET reconstruction, so this limitation no longer applies to these images.

Hybrid image reconstruction algorithms can be prone to artifacts where PET and MRI are misaligned, which in the torso may occur due to both bulk and physiological motion (36), as MR sequences often take less time to acquire than PET. Therefore, motion correction may also be required in addition to PSF modelling. However, HKEM has been demonstrated to be more robust to small misalignment between PET data and anatomical imaging than previous MR guided reconstruction algorithms (11, 12) due to the dependence of the kernel on PET iterative updates in addition to anatomical MR, and similarly the MR guided Q.Clear algorithm allows users to select appropriate weightings for both PET and MR image contributions to the penalisation term.

## 5 Conclusion

We have demonstrated that two algorithms, HKEM and the MR guided reconstruction, both lead to an increase in measured [^18^*F*]FDG PET tracer uptake in the spinal cord. However, comparison to ground-truth values on the XCAT phantom shows that bias remains large, indicating a need for further improvements in resolution recovery in quantitative PET-MRI of the spinal cord.

## Data Availability

The datasets presented in this article are not readily available because the data cannot be shared due to restrictions imposed by ethics approvals. Requests to access the datasets should be directed to s.sourbron@sheffield.ac.uk.
